# Identification and functional analysis of novel oncogene *DDX60L* in pancreatic ductal adenocarcinoma

**DOI:** 10.1186/s12864-021-08137-5

**Published:** 2021-11-18

**Authors:** Hongjin Wu, Weiwei Tian, Xiang Tai, Xuanpeng Li, Ziwei Li, Jing Shui, Juehua Yu, Zhihua Wang, Xiaosong Zhu

**Affiliations:** 1 The NHC Key Laboratory of Drug Addiction Medicine, The First Affiliated Hospital of Kunming Medical University, Kunming, Yunnan 650032 China; 2 International Research Center for Regenerative Medicine, BOAO International Hospital, Qionghai, Hainan 571434 China; 3 Yunnan Province Clinical Center for Bone and joint Diseases, The First Affiliated Hospital of Kunming Medical University, Kunming, Yunnan 650032 China; 4 Shanghai International Travel Healthcare Center, Shanghai, 200000 People’s Republic of China

**Keywords:** Pancreatic ductal adenocarcinoma (PDAC), Oncogene, DDX60L, MYEOV, CXCL2

## Abstract

**Background:**

Pancreatic ductal adenocarcinoma (PDAC) is a lethal cancer. Approximately 80% of patients initially diagnosed with locally advanced or metastatic disease survive only 4–11 months after diagnosis. Tremendous efforts have been made toward understanding the biology of PDAC.

**Results:**

In this study, we first utilized next-generation sequencing technique and existing microarray datasets to identify significant differentially expressed genes between PDAC and non-tumor adjacent tissue. By comparing top significant survival genes in PDAC Gene Expression Profiling Interactive Analysis database and PDAC transcriptome data from patients, our integrated analysis discovered five potential central genes (i.e., *MYEOV*, *KCNN4*, *FAM83A*, *S100A16*, and *DDX60L*). Subsequently, we analyzed the cellular functions of the potential novel oncogenes *MYEOV and DDX60L*, which are highly expressed in PDAC cells. Notably, the knockdown of *MYEOV and DDX60L* significantly inhibited the metastasis of cancer cells and induced apoptosis. Further RNA sequencing analyses showed that massive signaling pathways, particularly the TNF signaling pathway and nuclear factor-kappa B (NF-κB) signaling pathway, were affected in siRNA-treated cancer cells. The siDDX60L and siMYEOV significantly inhibited the expression of chemokine *CXCL2*, which may potentially affect the tumor microenvironment in PDAC tissues.

**Conclusions:**

The present findings identified the novel oncogene *DDX60L*, which was highly expressed in PDAC. Transcriptome profiling through siRNA knockdown of *DDX60L* uncovered its functional roles in the PDAC in humans.

**Supplementary Information:**

The online version contains supplementary material available at 10.1186/s12864-021-08137-5.

## Introduction

Pancreatic ductal adenocarcinoma (PDAC) is an extremely aggressive and deadly cancer. Approximately 80% of patients with PDAC are diagnosed with locally advanced or metastatic disease, and their prognosis remains dismal [1]. Genomic analysis of PDAC tissues showed that mutated cancer-related genes were significantly enriched, such as KRAS, TP53 [2]. These mutated genes aggregate into multiple signaling pathways, including KRAS, TGF-β, WNT, NOTCH [3]. Mutations in these genes will significantly change these signaling pathways, which play vital roles in regulating DNA repair, cell proliferation, cell survival and death, and further promote the progress of PDAC, including migration and metastasis [4, 5].

Currently, next-generation sequencing technologies are widely used for the identification of genetic alterations in cancer cells. Biological functions and molecular mechanisms of oncogenic genes and their interactions with cell signaling pathways involving cellular behaviors could also be revealed by advanced sequencing data analyses [6]. Thus far, a list of gene mutations and biomarkers, including serologic patterns, aberrant overexpressed mRNAs, miRNAs, proteins, and epigenetic signatures, have been associated with PDAC states. These could potentially be used as future early diagnostic and therapeutic strategies. In recent years, such strategies utilizing high-throughput next-generation sequencing discovered a series of novel somatic mutations and differentially expressed genes (DEGs) in solid tumors and circulating tumor cells [7–9]. However, the information obtained from genomic and transcriptomic sequencing data have not yet improved the care of patients with PDAC. The approach through which high-throughput genomic sequencing techniques can be applied to targeted-gene therapies for PDAC remains undetermined. Thus, there is an urgent need to identify novel oncogenes that can alter disease progression and provide guidance on therapeutic options for PDAC.

In the present study, we analyzed gene expression profiles using our RNA sequencing (RNA-seq) data and public microarray data of specimens obtained from patients with PDAC to identify highly interconnected genes that may serve as potential oncogenes for targeted therapy against PDAC. Furthermore, we investigated the function of these possible oncogenes in cell lines and revealed their associated signaling pathways that may be potentially used for targeted gene therapies.

## Materials and methods

### Cell culture

Human pancreatic cancer cell lines (MiaPaCa2 and PANC-1; American Type Culture Collection, Manassas, VA, USA) were maintained in Dulbecco’s modified Eagle’s medium supplemented with 2 mM glutamine, 1 mM Na-pyruvate, 100 units/ml penicillin, 100 μg/ml streptomycin, and 10% fetal bovine serum (all from Gibco, Thermo Fisher Scientific, Inc., Waltham, MA, USA) at 37 °C in a humidified atmosphere containing 10% CO_2_.

### Tissue collection, cell collection, and RNA extraction

Pancreatic cancer tissues were collected at The First Affiliated Hospital of Kunming Medical University (Kunming, China). Following resection, the tumor and adjacent tissues were examined by a pathologist, placed in cryotubes with RNAlater reagent, and frozen in liquid nitrogen. Total RNA was isolated using RNeasy Mini kit (Qiagen, Germantown, MD, USA) according to the instructions provided by the manufacturer. PANC-1 and MiaPaCa2 cells were transfected with siRNAs for 48 h (three replicates per sample) and collected. Total RNAs were extracted using the RNeasy Mini kit (Qiagen, Germantown, MD, USA). The quantity and quality of extracted RNAs were assessed using a Nanodrop spectrophotometer and Agilent Bioanalyzer 2100 (Agilent Technologies, Inc., Santa Clara, CA, USA), respectively. Sequence libraries were prepared using a TruSeq Stranded mRNA Library Prep kit for NeoPrep, according to the instructions provided by the manufacturer, and sequenced using an Illumina HiSeq 2000 platform. All the RNA-seq data in the present study are available from the Gene Expression Omnibus repository (GSE171485 and GSE171486).

### RNA-seq and bioinformatics analyses

Sequencing libraries were constructed and sequenced using the Illumina HiSeq2000 platform with SE50. A total of 21.0 ± 6.9 million reads were generated for each sample. RNA-seq data were aligned to the human reference genome (GrCH37, Ensembl build 74) using Tophat version 2.0.12 [10]. Gene expression levels, represented as fragments per kilo-base per million mapped reads (FPKM), were obtained for 63,783 genes/transcripts annotated using Ensemble GrCH37 database release 74. Functional and pathway enrichments were assessed using the Database for Annotation, Visualization and Integrated Discovery (DAVID) bioinformatics resources. Only functional/pathway enrichments meeting a false-discovery rate < 5% are presented.

### Public databases used in this study

For identifying the potential oncogenes in PDAC, we downloaded Affymetrix CEL files from the Gene Expression Omnibus database with accession number GSE28735, GSE16535, and GSE15471. For gene expression in cancer tissues, we downloaded the expression data in Gene Expression Profiling Interactive Analysis (GEPIA, http://gepia.cancer-pku.cn/). For gene expression in cancer cell lines, we downloaded the expression data in Cancer Cell Line Encyclopedia (CCLE, https://depmap.org/portal/download/) Expression 21Q2 Public.

### Microarray data analysis

The microarray gene expression data were processed and analyzed using R and Bioconductor. Affymetrix U133 plus 2.0 whole genome microarrays and Affymetrix Gene Chip Human Gene ST1.0 microarrays were analyzed using R with affy and oligo packages, respectively. The microarray data were subsequently normalized using the Robust Multi-Array Average RMA method. The normalized expression values from all samples were log2 transformed. Genes differentially regulated between the biologic groups were identified using *limma*.

### Quantitative real-time polymerase chain reaction (PCR)

For quantitative real-time PCR, 1 μg of total RNA was reverse-transcribed and triplicate PCR reactions were performed on an ABI 7500 Real-Time PCR System (Foster City, CA, USA). Glyceraldehyde-3-phosphate dehydrogenase mRNA was used as internal control. The PCR reaction was conducted according to the recommended protocol. The primers used for quantitative PCR are described in Supplementary Table S1.

### Cell viability assay

Cell viability was determined using the Cell Counting Kit-8 (CCK-8) assay. Briefly, fresh culture medium containing 10 μl CCK-8 (Dojindo Molecular Technologies, Inc.) was placed in each well. The culture plates were incubated for an additional 4 h at 37 °C, and absorbance was measured at 450 nm using a microplate spectrophotometer (Molecular Devices Corp., Sunnyvale, CA, USA).

### Cell Transwell assay

The chambers were placed in a 24-well plate; serum-free medium (100 μl) was added to the upper chamber, and the plate was placed in an incubator at 37 °C for 1 h. A serum-free cell suspension was prepared, and 100 μl (10^5^ cells) were placed on the plate. Next, 30% fetal bovine serum medium (600 μl) was added in the lower chamber, and the cells were incubated at 37 °C. The cells were fixed with 4% paraformaldehyde. The cells were counted using microscope photos (original magnification × 200), the data were analyzed, and differences in cell migratory ability between the experimental group (siRNAs) and the control group (siControl) were determined.

### The Kaplan–Meier plotter analysis

To explore the potential prognostic value of the genes in cancer patients, the Kaplan–Meier Plotter analysis (http://kmplot.com/analysis/) pan-cancer RNA-seq database was used to perform overall survival analysis (PDAC, *n* = 177) [11]. In accordance with the instructions, the prognostic value of two genes were also analyzed with the Kaplan–Meier Plotter (PDAC), and GraphPad Prism 8.3.0 was used to further display the data.

### Statistical analysis

All experiments in this study were independently performed in triplicate. The data are presented as the mean with standard error of the mean or standard deviation. All graphs were plotted and analyzed using the GraphPad Prism 8 software (San Diego, CA, USA). A *p*-value < 0.05 denoted statistically significant difference.

## Results

### PDAC DEGs identified from RNA-seq and microarray analyses

**Fig. 1:**
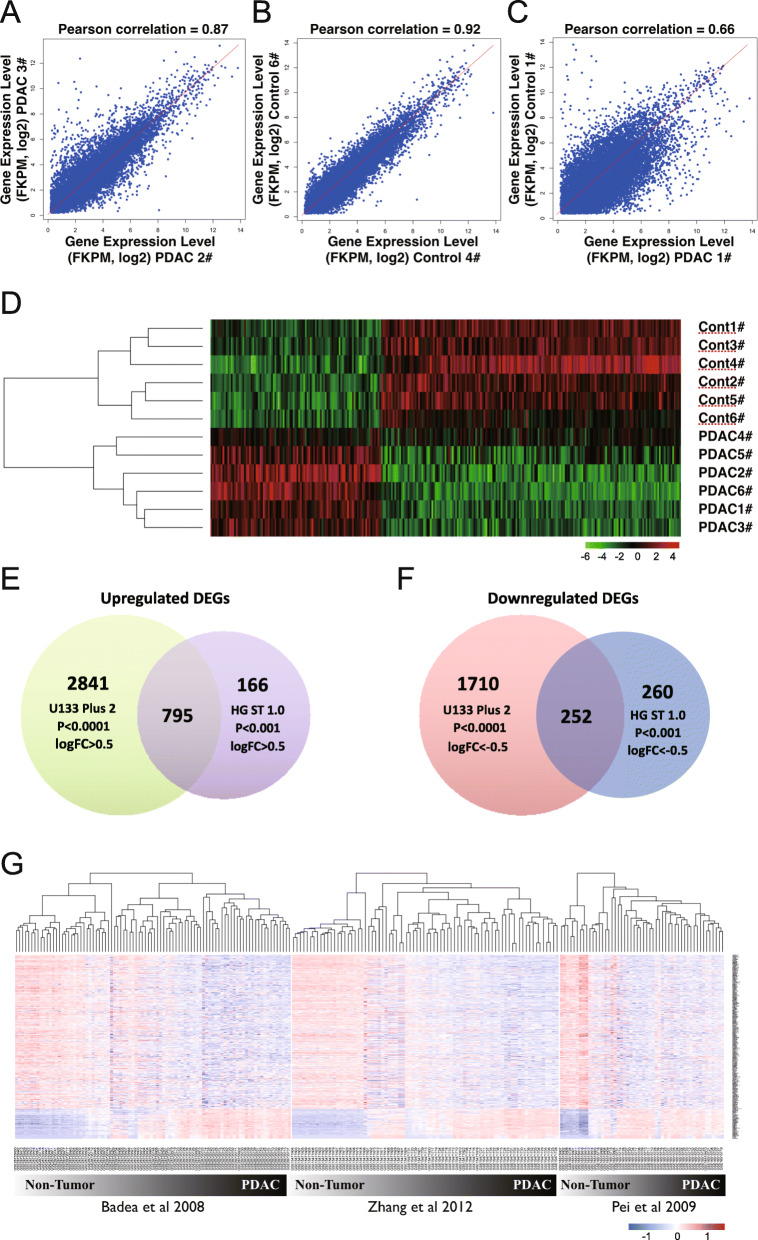
Identification of differentially expressed genes (DEGs) in PDAC using RNA-seq and microarray data. (**A–C**) Scatter plots of FPKM values for gene expression of PDAC versus PDAC (**A**), non-tumor pancreas versus non-tumor pancreas (**B**), and PDAC versus matching non-tumor pancreas (**C**). Pearson correlation coefficients are shown above the corresponding scatter plots. (**D**) Heatmap of 1371 DEGs identified from the comparison between six PDAC and six non-tumor control specimens (*p* < 0.05 and |log2FC| > 0.5). (**E, F**) Venn diagram of DEGs identified from previous published microarray studies of PDAC versus non-tumor controls using two types of Affymetrix microarray platforms. Overlapping upregulated DEGs (*n* = 795) (**E**) and downregulated DEGs (*n* = 252) (**F**) are shown. (**G**) Heatmaps and hierarchical clustering of all PDAC specimens versus non-tumor controls from three independent groups clearly show that these 1047 DEGs can distinguish PDAC from non-tumor pancreas specimens FC, fold change; FPKM, fragments per kilo-base per million mapped reads; PDAC, pancreatic ductal adenocarcinoma; RNA-seq, RNA sequencing.

For the identification of specific genes expressed in PDAC, we constructed sequencing libraries from polyadenylated-RNA extracted from six PDAC specimens, three non-tumor adjacent tissues, and three pancreatic tissues from normal individuals. Approximately 21 M raw reads were obtained. We aligned the RNA-seq data, yielding an average mapping rate of 97.0 ± 0.6%. The quality of the RNA-seq data was determined using the Pearson correlation of the associations between the transcriptome data obtained from different specimens. RNA-seq data indicated that the Pearson correlation coefficients of transcriptome data with same phenotypes (PDAC versus PDAC: 0.87 and Control versus Control: 0.92) were higher than those noted with different phenotypes (PDAC versus Control: 0.66) (Figs. 1A–C). These findings suggested that non-tumor pancreas and PDAC specimens show distinct global gene expression patterns. The heatmap of a total of 1371 unique DEGs identified from the comparison between six PDAC and six non-tumor control pancreas specimens distinguished PDAC versus non-tumor controls (Fig. 1D). Of those, 607 genes were upregulated and 763 genes were downregulated in PDAC specimens using cutoff criteria of |log2(fold-change [FC])| > 0.5 (log2(FC) > 0.5) and *p* < 0.05 (the full list of DEGs is provided in Supplementary Table S2A).

AffymetrixHG-U133 Plus 2.0 and Human Gene 1.0 ST microarray platforms have been commonly used in previous studies [12–14] to study the gene expression profiles of the whole genome. This approach resulted in the discovery novel tumor biomarker genes and investigated the molecular mechanisms for PDAC [15]. We sought to better understand the biology of cancer and compare the numbers and percentages of overlapping DEGs between RNA-seq and other independent data sets on microarray platforms. For this purpose, we revisited the gene expression profiles of PDAC versus non-tumor pancreatic tissues by reprocessing the gene expression microarray data from three independent studies [12–14]. We combined two data sets on the same Affymetrix HG-U133 Plus 2.0 platform and processed the combined data sets (GSE15471 and GSE16515) [12, 13] using R and Bioconductor packages “affy” and “affycoretools”. In the comparison between 75 PDAC and 55 non-tumor pancreatic tissue gene expression profiles, 7776 probes which reflect a total of 5598 unique genes (log2(FC) > 0.5 and *p* < 0.001) were revealed to be differentially expressed between PDAC and non-tumor pancreas tissues. The identified DEGs consist of 3636 unique upregulated genes (5388 probes) and 1962 unique downregulated genes (2388 probes) in PDAC (the full list of DEGs is provided in Supplementary Table S2B). Meanwhile, using R and the Bioconductor package “oligo”, we obtained a list of unique DEGs by reanalyzing the dataset GSE28735 of 90 human PDAC and adjacent non-tumor tissues (45 PDAC versus 45 non-tumor specimens) reported by Zhang [14] using the Affymetrix Human Gene 1.0 ST microarray platform. A total of 1546 probes (1473 unique DEGs) were identified; 1006 probes (961 unique genes) were upregulated and 540 probes (512 unique genes) were downregulated in PDAC with cutoff criteria of |log2(FC)| > 0.5 and *p* < 0.001 (the full list of DEGs is provided in Supplementary Table S2C).

To determine which overrepresented signaling pathways are shared among all PDAC data sets, we compared the Kyoto Encyclopedia of Genes and Genomes (KEGG) pathways identified from all lists of DEGs. DAVID-KEGG analysis of these DEGs revealed that the shared signaling pathways for upregulated genes across all data sets at three platforms were the signaling pathways of the pathways in cancer, p53, extracellular matrix-receptor, apoptosis, and cell cycle (Supplementary Table S3). However, there were no shared KEGG pathways identified for downregulated genes in the DEG lists in these data sets.

### Identification of potential oncogenes responsible for the progression of PDAC

We subsequently compared the lists of DEGs identified from the data sets on two independent Affymetrix microarray platforms. Approximately 82% (795/961) of the upregulated DEGs and 49% (252/512) of the downregulated DEGs from Human Gene 1.0 ST microarray data were also identified in the HG-U133 Plus 2.0 microarray platform (Fig. 1E and F). Heatmaps and hierarchical clustering of all PDAC versus non-tumor pancreatic specimens obtained from three independent groups clearly showed that these 1047 DEGs can distinguish between PDAC and non-tumor pancreas specimens (Fig. 1G). With higher statistical stringency, the comparison between two lists of DEGs from microarray data revealed 204 upregulated and 39 downregulated overlapped DEGs in PDAC using cutoff criteria of |log2(FC)| > 1 and *p* < 0.0001 (the full lists of overlapped DEGs are provided in Supplementary Table S4A).

**Fig. 2:**
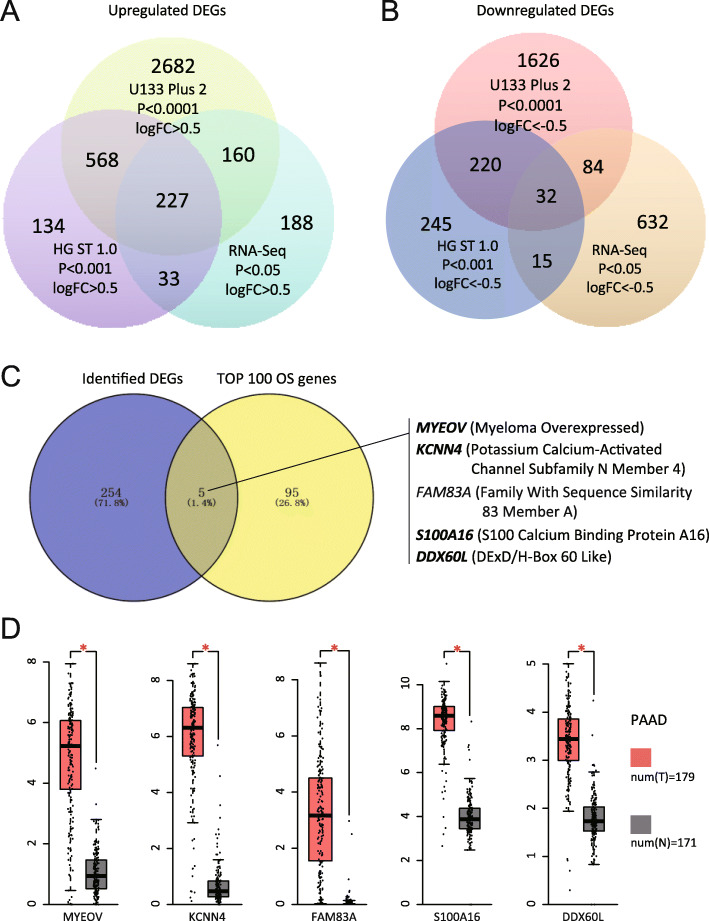
Identification of overlapped genes responsible for the progression of PDAC. (**A-B**) Venn diagram of DEGs identified in the RNA-seq analysis of this study and in previous microarray studies of PDAC versus non-tumor controls. Overlapping upregulated (**A**) and downregulated (**B**) DEGs across multiple platforms are shown. (**C**) Venn diagram of the identified DEGs and the Top 100 overall survival-related genes in PDAC. Five most significant potential oncogenes were identified: *MYEOV*, *KCNN4*, *FAM83A*, *S100A16*, and *DDX60L*. (**D**) Expression of five significant potential oncogenes in PDAC tissues of the GEPIA database. One-way ANOVA is used to analyze significant differences in the gene expression levels between PDAC normal tissues and cancer tissues. *represents significant differences in RNA expression, *p* < 0.01. *DDX60L*, DExD/H-box 60 like; DEGs, differentially-expressed genes; *FAM83A*, family with sequence similarity 83 member A; GEPIA, Gene Expression Profiling Interactive Analysis; *KCNN4*, potassium calcium-activated channel subfamily N member 4; *MYEOV*, myeloma overexpressed; PDAC, pancreatic ductal adenocarcinoma; RNA-seq, RNA sequencing; *S100A16*, S100 calcium binding protein A16

Next, we compared the list of DEGs from our PDAC RNA-seq data with the lists of DEGs identified from the above microarray data sets. The cross-platform comparison revealed that approximately 43% (260/607) and 64% (387/607) of the upregulated DEGs identified through RNA-seq overlapped with the lists of DEGs obtained from the HG 1.0 ST and HG-U133 Plus 2.0 platforms, respectively (Fig. 2A). Conversely, only 6% (47/764) and 15% (116/764) of the downregulated DEGs recorded from the RNA-seq data were identified from the HG 1.0 ST and HG-U133 Plus 2.0 data sets, respectively (Fig. 2B). The cross-platform analysis identified 227 upregulated and 32 downregulated genes in PDAC that were shared in both RNA-seq data and two other microarray data sets (the full list of DEGs is provided in Supplementary Table S4B).

For the identification of the most significant potential oncogenes in PDAC, we overlapped these 259 identified genes with the top 100 significant survival genes in PDAC (Gene Expression Profiling Interactive Analysis [GEPIA] database); five genes (myeloma overexpressed [*MYEOV*], potassium calcium-activated channel subfamily N member 4 [*KCNN4*], family with sequence similarity 83 member A [*FAM83A*], S100 calcium binding protein A16 [*S100A16*], and DExD/H-box 60 like [*DDX60L*]) were identified (Fig. 2C, Supplementary Table S4C). Kaplan–Meier Plotter analysis showed that the expression levels of these five genes are also closely related to the prognosis of patients with PDAC. Of note, patients with pancreatic cancer expressing high levels of these potential oncogenes have a very poor prognosis (*p* < 0.001) (Supplementary Fig. S1A). In the GEPIA database, these potential oncogenes were highly expressed in the cancer tissues of patients with pancreatic cancer (Fig. 2D), and their expression levels were positively correlated with the cancer stages (Supplementary Fig. S1B). Compared with the early-stage (stages I–II), pancreatic cancer tissues obtained from patients with stage III–IV disease showed markedly higher expression levels of these potential oncogenes. The results indicated that these genes may be involved in the progression of pancreatic cancer (Supplementary Fig. S1B). In summary, based on our RNA-seq and public microarray data, we identified five potential oncogenes that may play vital roles in the occurrence and progression of PDAC.

### Expression and siRNA knockdown of novel oncogene DDX60L in PDAC

**Fig. 3:**
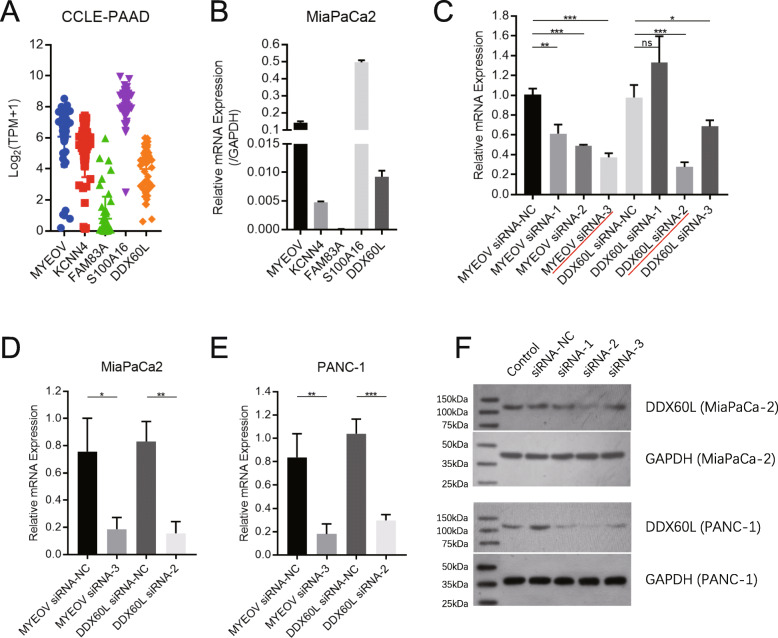
Knockdown of *MYEOV*, *KCNN4*, *S100A16*, and *DDX60L* expression through siRNA. (**A**) Expression of *MYEOV*, *KCNN4*, *FAM83A*, *S100A16*, and *DDX60L* in pancreatic cancer cells using data from the CCLE database (CCLE-PAAD). (**B**) Expression of *MYEOV*, *KCNN4*, *FAM83A*, *S100A16* and *DDX60L* in MiaPaca2 pancreatic cancer cell (compared with the expression of GAPDH). (**C**) Screening for siRNAs that could knock down the expression of *MYEOV* and *DDX60L* in MiaPaca2 cells. (**D, E**) Knockdown of *MYEOV* and *DDX60L* expression with indicated siRNAs in MiaPaca2 cells (**D**) and PANC-1 cells (**E**)

We investigated the expression of these potential oncogenes (*MYEOV*, *KCNN4*, *FAM83A*, *S100A16*, and *DDX60L*) in the pancreatic cancer cell lines of the Cancer Cell Line Encyclopedia (CCLE) database. The results showed that *MYEOV*, *KCNN4*, *S100A16*, and *DDX60L* are highly expressed in most pancreatic cancer cell lines, but *FAM83A* is not (Fig. 3A). Further quantitative real-time-PCR validation performed using the MiaPaCa2 cell line was consistent with the CCLE data (Fig. 3B). Recent studies have shown that *MYEOV* [16–18], *KCNN4* [19, 20], and *S100A16* [21–23] act as oncogenes and play vital roles in cancer progression. In this study, we found a novel potential oncogene – *DDX60L*. To validate the potential functions of *DDX60L* in pancreatic cancer (*MYEOV* was used as positive control), we designed three siRNAs to knockdown its expression in cancer cells (sequences are shown in Supplementary Table S1B). The knockdown efficiency of these siRNAs was tested using the MiaPaCa2 cell line. The analysis showed that siMYEOV-3 and siDDX60L-2 resulted in effective inhibition of target gene expression (Fig. 3C). Next, we used these validated siRNAs to inhibit the expression of target genes in MiaPaCa2 and PANC-1 cells, demonstrating excellent effectiveness (> 60%) (Fig. 3D and E).

One-way ANOVA is used to analyze significant differences in the gene expression levels between control cancer cells and siRNA treated cancer cells. *p < 0.05; **p < 0.01; ***p < 0.001; ***p < 0.0001. CCLE, Cancer Cell Line Encyclopedia; *DDX60L*, DExD/H-box 60 like; *FAM83A*, family with sequence similarity 83 member A; GAPDH, glyceraldehyde-3-phosphate dehydrogenase; *KCNN4*, potassium calcium-activated channel subfamily N member 4; *MYEOV*, myeloma overexpressed; PAAD, pancreatic adenocarcinoma; *S100A16*, S100 calcium binding protein A16.

### Knockdown of DDX60L inhibited the migration of PDAC cells, but not their proliferation

**Fig. 4:**
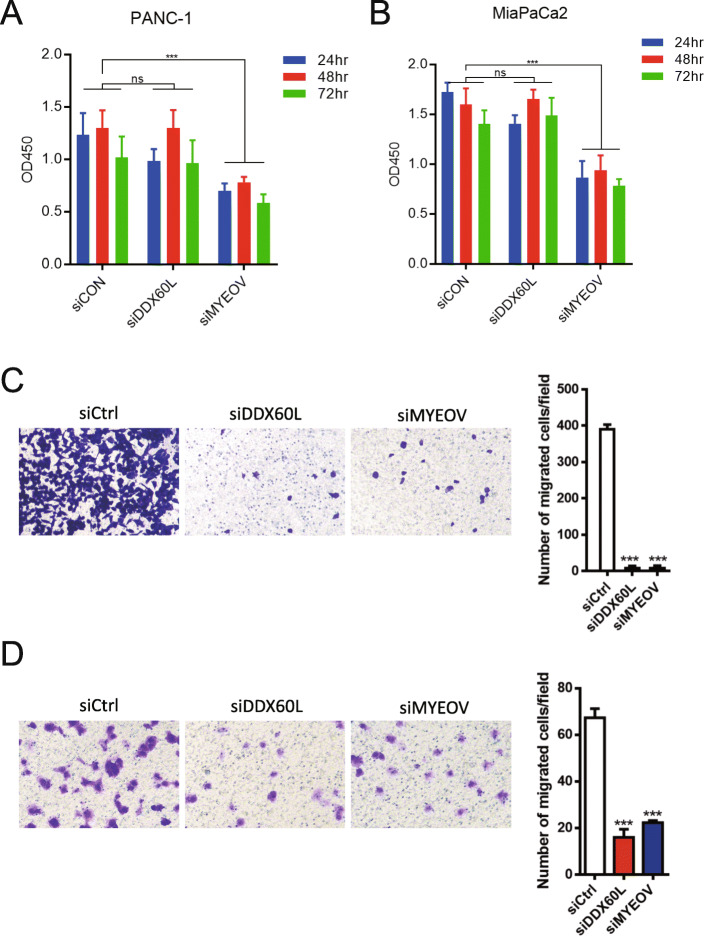
Knockdown of *DDX60L* inhibited the migration of PDAC cells, but not their proliferation. (**A, B**) Viability of siRNA-transfected PDAC cells. CCK-8 assay analyzed the siControl-, siMYEOV-, and siDDX60L-transfected PANC-1 (**A**) and MiaPaca2 (**B**) cells. (**C, D**) The invasive ability of siRNA-transfected PDAC cells. Transwell assay analyzed the siControl-, siMYEOV-, and siDDX60L-transfected PANC-1 (**C**) and MiaPaca2 (**D**) cells. One-way ANOVA is used to analyze significant differences. *p < 0.05; **p < 0.01; ***p < 0.001. CCK-8, Cell Counting Kit-8; *DDX60L*, DExD/H-box 60 like; MYEOV, myeloma overexpressed; PDAC, pancreatic ductal adenocarcinoma

*DDX60L* is highly expressed in pancreatic cancer and related to the overall survival of patients with cancer. Therefore, it may play a vital role in the progression of pancreatic cancer. Using siRNA knockdown of *DDX60L*, we further investigated its potential functions in MiaPaCa2 and PANC-1 cells. The CCK-8 assay of siRNA knockdown target genes showed that siMYEOV significantly reduced the proliferation of MiaPaCa2 and PANC-1 cells (*p* < 0.001), whereas siDDX60L had no effect on proliferation (Fig. 4A and B). The effect of *DDX60L* on the migration of pancreatic cancer cells was investigated using Transwell migration assay. The results showed that siRNA knockdown of *MYEOV* and *DDX60L* could significantly inhibit the migration of MiaPaCa2 and PANC-1 cells (Fig. 4C and D).

### Knockdown of DDX60L induced apoptosis of PDAC cells, but not cell cycle arrest

**Fig. 5:**
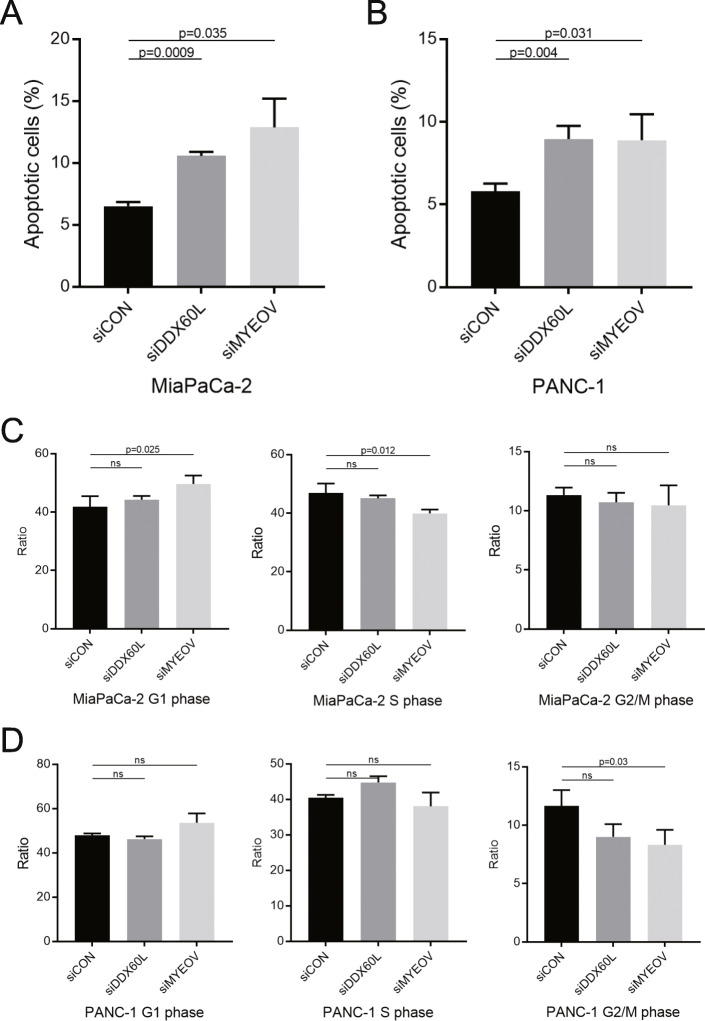
Knockdown of *DDX60L* induced apoptosis of PDAC cells, but not cell cycle arrest. (**A, B**) Apoptosis of siRNA-transfected PDAC cells. FACS analyzed the apoptosis of the siControl-, siMYEOV-, and siDDX60L-transfected PANC-1 (**A**) and MiaPaca2 (**B**) cells. (**C, D**) Cell cycle of siRNA-transfected PDAC cells. FACS analyzed the cell cycle of the siControl-, siMYEOV-, and siDDX60L-transfected PANC-1 (**C**) and MiaPaca2 (**D**) cells. One-way ANOVA is used to analyze significant differences. Ns, no significant differences, *p < 0.05; **p < 0.01; ***p < 0.001. *DDX60L*, DExD/H-box 60 like; FACS, fluorescence-activated cell sorting; MYEOV, myeloma overexpressed; PDAC, pancreatic ductal adenocarcinoma

Since *DDX60L* did not affect the proliferation of pancreatic cancer cells, we also investigated the rate of apoptosis and cell cycle in pancreatic cancer cells. The analysis was performed in siRNA-treated or -untreated pancreatic cancer cells through flow cytometry. The apoptosis analysis of siRNA-treated MiaPaCa2 and PANC-1 cells showed that, compared with control siRNA, siDDX60L (*p* < 0.001 in MiaPaCa2; *p* < 0.01 in PANC-1 cells) and siMYEOV (*p* < 0.05 in MiaPaCa2; *p* < 0.05 in PANC-1 cells) significantly induced cell apoptosis (Fig. 5A and B). The cell cycle analysis of siRNA-treated MiaPaCa2 and PANC-1 cells showed that, compared with control siRNA, siDDX60L and siMYEOV did not have any significant effects on the cell cycle (Fig. 5C and D). In summary, these results indicated that *DDX60L* could inhibit the migration of pancreatic cancer cells and induce their apoptosis, which may play a vital role in the progression of pancreatic cancer.

### Transcriptome analyses revealed alterations of genes and signaling pathways in PDAC after knockdown of DDX60L

The transcriptome alterations induced by siDDX60L were also investigated through RNA-seq. For the transcriptome analysis, we acquired approximately 26 M clean reads from siRNA-transfected and control MiaPaCa2 and PANC-1 cells with Q30 > 90%, respectively. After bioinformatics analysis of these RNA-seq data, the upregulated and downregulated DEGs were identified.

**Fig. 6:**
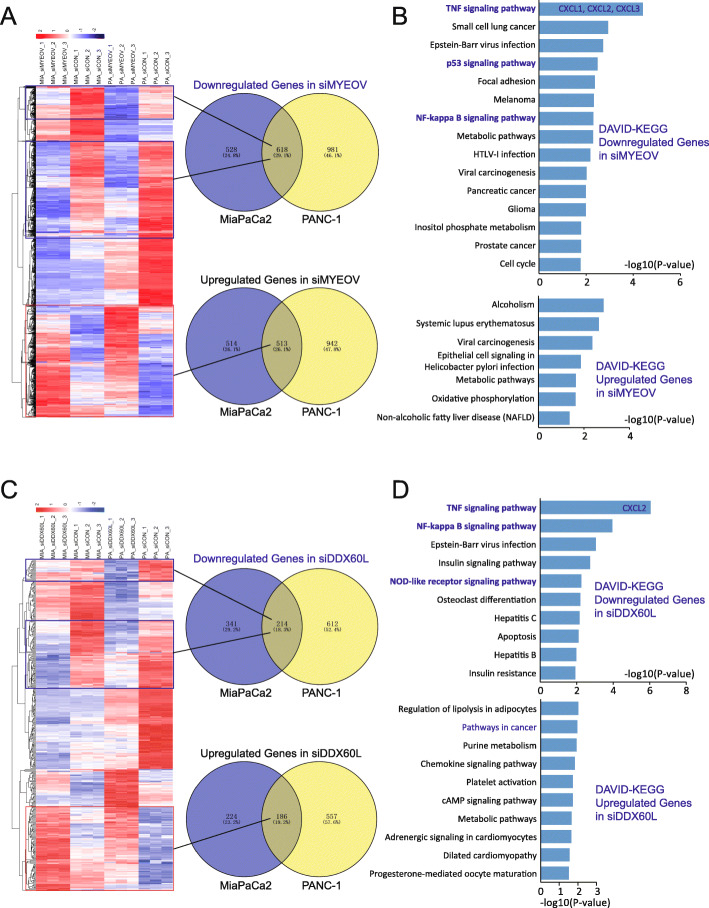
Transcriptome analyses showed alterations of genes and signaling pathways in siMYEOV- and siDDX60L-transfected PDAC cells. (**A**) Heatmap and Venn diagram of DEGs identified in siMYEOV-transfected MiaPaca2 and PANC-1 cells. (**B**) DAVID-KEGG analysis of the DEGs in siMYEOV-transfected MiaPaca2 and PANC-1 cells (*p* < 0.05). (**C**) Heatmap and Venn diagram of DEGs identified in siDDX60L-transfected MiaPaca2 and PANC-1 cells. (**D**) DAVID-KEGG analysis of the DEGs in siDDX60L-transfected MiaPaca2 and PANC-1 cells (*p* < 0.05). DAVID-KEGG, Database for Annotation, Visualization and Integrated Discovery-Kyoto Encyclopedia of Genes and Genomes; DDX60L, DExD/H-box 60 like; DEGs, differentially-expressed genes; PDAC, pancreatic ductal adenocarcinoma; MYEOV, myeloma overexpressed

For the positive control gene, MiaPaCa2 and PANC-1 cells were transfected with siMYEOV; 618 downregulated genes and 513 upregulated genes were identified (Fig. 6A). The KEGG_PATHWAY category of these DEGs showed that the upregulated genes were clustered into seven subcategories (*p* < 0.05; three subcategories with *p* < 0.01), including alcoholism (*p* = 0.0014), systemic lupus erythematosus (*p* = 0.0022), and viral carcinogenesis (*p* = 0.0043) (Fig. 6B, Supplementary Table S5A). The downregulated genes were clustered into 27 subcategories (*p* < 0.05; 10 subcategories with *p* < 0.01), including the tumor necrosis factor (TNF) signaling pathway (*p* = 3.92*E-05), small cell lung cancer (*p* = 0.0012), p53 signaling pathway (*p* = 0.003), nuclear factor-kappa B (NF-κB) signaling pathway (*p* = 0.005) and cell cycle (*p* = 0.017) (Fig. 6B, Supplementary Table S5A). In particular, siMYEOV significantly inhibited the expression of chemokine C-X-C motif chemokine ligand 1 (*CXCL1*), chemokine C-X-C motif chemokine ligand 2 (*CXCL2*), and chemokine C-X-C motif chemokine ligand 3 (*CXCL3*) in the TNF signaling pathway. These results suggested that siRNA knockdown of *MYEOV* repressed the expression of genes involved in the cell cycle, and significantly influenced the immune response of PDAC cells.

In siDDX60L-transfected MiaPaCa2 and PANC-1 cells, 214 downregulated genes and 186 upregulated genes were identified (Fig. 6C). These DEGs were subjected to DAVID analysis (a web-based high-throughput functional genomics analysis tool) for a systematic clustering of these genes. In the KEGG_PATHWAY category, we found that the upregulated genes were clustered into 16 subcategories (*p* < 0.05; one subcategory with *p* < 0.01), including the pathways in cancer (*p* = 0.011) and purine metabolism (*p* = 0.012) (Fig. 6D, Supplementary Table S5B). The downregulated genes were clustered into 15 subcategories (*p* < 0.05; eight subcategories with *p* < 0.01), including the TNF signaling pathway (*p* = 8.91*E-07), NF-κB signaling pathway (*p* = 0.0001), insulin signaling pathway (*p* = 0.0018), and nucleotide-binding oligomerization domain (NOD)-like receptor signaling pathway (*p* = 0.0055) (Fig. 6D, Supplementary Table S5B). Interestingly, siDDX60L significantly inhibited the expression of chemokine *CXCL2* in the TNF signaling pathway. These results indicate that siRNA knockdown of DDX60L significantly altered the TNF and NF-κB signaling pathways in PDAC cells. Hence, the downregulated chemokines may influence the immune response of PDAC cells.

### Knockdown of DDX60L and MYEOV inhibited the expression of CXCL2 and potentially affected the prognosis in PDAC

**Fig. 7:**
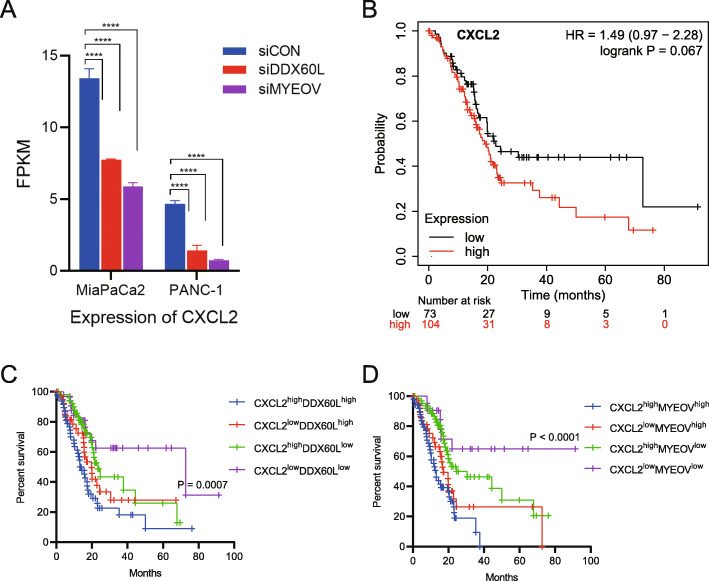
Knockdown of *DDX60L* and *MYEOV* inhibited the expression of *CXCL2* and led to good prognosis in PDAC. (**A**) Expression of *CXCL2* in siDDX60L- and siMYEOV-transfected MiaPaCa2 and PANC-1 cells. Two-way ANOVA is used to analyze significant differences in the gene expression levels between control cancer cells and siRNA treated cancer cells. ****p < 0.0001. (**B**) The Kaplan–Meier Plotter analysis showed that patients from the TCGA-PDAC dataset (*n* = 177) [11] who expressed high levels of *CXCL2* had a poor prognosis. (**C-D**) The Kaplan–Meier Plotter analysis showed that PDAC patients with low *CXCL2* and *DDX60L* expression in tumors (**C**) and those with low *CXCL2* and *MYEOV* expression in tumors (**D**) had a significantly improved overall survival. The *p*-values are shown. *CXCL2*, C-X-C motif chemokine ligand 1; *DDX60L*, DExD/H-box 60 like; *MYEOV*, myeloma overexpressed; OS, overall survival; PDAC, pancreatic ductal adenocarcinoma; TCGA, The Cancer Genome Atlas

Interestingly, the RNA-seq data of *DDX60L* and *MYEOV* showed some similar alterations of the signaling pathways in PDAC cells, such as the TNF signaling pathway and NF-κB signaling pathway. These pathways were significantly inhibited in PDAC cells treated with siDDX60L and siMYEOV. Similarly, we found that the expression of neutrophil chemotactic chemokine *CXCL2* was significantly inhibited in PDAC cells treated with siDDX60L and siMYEOV (Fig. 7A). With the Kaplan–Meier Plotter analysis, the expression of *CXCL2* in pancreatic cancer significantly affects the prognosis of patients (*n* = 177), and high expression levels are associated with a poor prognosis (Fig. 7B). Furthermore, by combining the expression levels of *DDX60L* and *MYEOV*, the cohorts of CXCL2^low^DDX60L^low^ and CXCL2^low^MYEOV^low^ showed a better prognosis in patients with PDAC from TCGA database (Fig. 7C and D). In summary, these data indicated that the four genes identified in this study affect the cellular functions of PDAC. These genes may also affect the microenvironment of cancer cells through transcriptomic alterations of chemokines.

## Discussion

In this study, we integrated a large number of transcriptome profiling data from PDAC and non-tumor pancreatic tissue specimens to identify significant differentially expressed genes. A list of aberrantly overexpressed genes was associated with PDAC states. Among these identified genes, we found five genes related to cancer prognosis, and further verified their cellular functions and potential signaling pathways in pancreatic cancer cells. Therefore, it is reasonable to hypothesize that these genes could be served as immunohistochemical prognostic markers and potential therapeutic targets for PDAC.

Interestingly, the five genes identified in this study (*DDX60L*, *FAM83A*, *KCNN4*, *MYEOV*, and *S100A16*) showed some similar characteristics; they were highly expressed and corelated with poor prognosis in pancreatic cancer. Previously, *MYEOV* [16–18], *KCNN4* [19, 20], *S100A16* [21–23], and *FAM83A* [24, 25] had been reported as oncogenes in various types of cancer, which could promote the proliferation and invasion of cancer cells. However, the novel oncogene *DDX60L* we identified did not influence the proliferation of cancer cells, but significantly influenced their invasive ability. Further RNA-seq analysis showed that siRNA knockdown of *DDX60L* significantly inhibited the genes in the TNF signaling and NF-κB signaling pathway, particularly the chemotactic cytokine CXCL2.

Chemotactic cytokines, also termed chemokines, play a key role in mediating the recruitment of immune cells to tumor sites [26, 27]. CXC chemokine CXCL2 possesses potent neutrophil chemotactic activity [28, 29]. The involvement of tumor monocyte-derived chemokines and cytokines in modulating neutrophil accumulation and functions was previously studied [30, 31]. The knockdown of oncogenes *MYEOV* and *DDX60L* inhibited the expression of *CXCL2* in pancreatic cancer cells. These findings suggested that repression of these oncogenes could potentially reduce the metastasis of cancer cells and repress the immunosuppressive ability of cancer cells. However, these results should be further validated in mouse xenograft models of pancreatic cancer.

## Conclusions

The present study integrated a systematic approach to identify key genes associated with PDAC tumorigenesis based on gene expression profiling data. Several key genes including *MYEOV*, *KCNN4*, *FAM83A*, *S100A16*, and *DDX60L* were identified. Further cellular experiments validated the function of the novel oncogene *DDX60L*. These genes could potentially serve as targets and for tumor imaging to guide therapeutic selection in PDAC.

## Supplementary Information

**Additional file 1: Fig. S1** RNA expression levels of the five key genes and their relationship with overall survival in PDAC. (A) Relationship of the five key genes with overall survival in PDAC. (B) RNA expression of the five key genes in PDAC. PDAC, pancreatic ductal adenocarcinoma

**Additional file 2: Fig. S2.** Influence of *MYEOV* and *DDX60L* in the cell cycle of PDAC. *DDX60L*, DExD/H-box 60 like; *MYEOV*, myeloma overexpressed; PDAC, pancreatic ductal adenocarcinoma

**Additional file 3: Supplementary Table S1.** The qRT-PCR primers and siRNAs used in this study. qRT-PCR, quantitative real-time-polymerase chain reaction

**Additional file 4: Supplementary Table S2.** DEGs of human PDAC and adjacent non-tumor tissues identified using our RNA-seq data (2A), as well as the GSE15471 and GSE16515 (2B), and GSE28735 (2C) data sets. DEGs, differentially-expressed genes; RNA-seq, RNA sequencing

**Additional file 5: Supplementary Table S3.** DAVID-KEGG analyses of upregulated genes in our RNA-seq and published microarray data. DAVID-KEGG, Database for Annotation, Visualization and Integrated Discovery-Kyoto Encyclopedia of Genes and Genomes; RNA-seq, RNA sequencing

**Additional file 6: Supplementary Table S4.** Venn diagram of overlapped DEGs in the microarray and RNA-seq platform analyses of PDAC and the top 100 survival OS-related genes of PDAC. (4A) Overlapped DEGs in the microarray platform of PDAC using cutoff criteria of |log2(FC)| > 1 and *p* < 0.0001. (4B) Overlapped DEGs in our RNA-seq data and three published microarray data. (4C) Top 100 OS-related genes of PDAC. DEGs, differentially-expressed genes; OS, overall survival; PDAC, pancreatic ductal adenocarcinoma; RNA-seq, RNA sequencing

**Additional file 7: Supplementary Table S5.** DAVID analysis of upregulated and downregulated genes in siMYEOV- (5A) and siDDX60L- (5B) transfected MiaPaCa2 and PANC-1 cells. DAVID, Database for Annotation, Visualization and Integrated Discovery; DDX60L, DExD/H-box 60 like; MYEOV, myeloma overexpressed

## Data Availability

All the RNA-seq data in the present study are available from the Gene Expression Omnibus repository GSE171485 (https://www.ncbi.nlm.nih.gov/geo/query/acc.cgi?acc=GSE171485) and GSE171486 (https://www.ncbi.nlm.nih.gov/geo/query/acc.cgi?acc=GSE171486).
